# Nationwide Analysis of PCI After TAVR From the Netherlands Heart Registration

**DOI:** 10.1002/ccd.70428

**Published:** 2025-12-10

**Authors:** Hugo M. Aarts, Kimberley I. Hemelrijk, Gijs M. Broeze, Steven A. Muller, Lineke Derks, Ronak Delewi, Michiel Voskuil, the Transcatheter Heart Intervention and Percutaneous Coronary Intervention registration committees of the Netherlands Heart Registration

**Affiliations:** ^1^ Department of Cardiology University Medical Center Utrecht Utrecht The Netherlands; ^2^ Department of Cardiology Amsterdam University Medical Center Amsterdam The Netherlands; ^3^ Netherlands Heart Registration Utrecht The Netherlands

**Keywords:** coronary access, percutaneous coronary intervention, transcatheter aortic valve replacement

## Abstract

**Background:**

Percutaneous coronary intervention (PCI) after transcatheter aortic valve replacement (TAVR) has gained interest as concomitant coronary artery disease (CAD) is now often treated conservatively before TAVR, and TAVR is increasingly used in younger patients with longer life expectancies. Therefore, more contemporary data on PCI after TAVR are warranted to optimize CAD treatment and guide lifetime management. The primary objective was to evaluate the incidence of PCI in patients with prior TAVR, including insights on trends and procedural and clinical outcomes from a large, nationwide cohort.

**Methods:**

Data from the Netherlands Heart Registration were used to identify patients with prior TAVR who underwent PCI between January 2015 and September 2021.

**Results:**

Among 216,813 PCI patients, 419 (0.19%) had previously undergone TAVR, representing an incidence of 2.81% among all TAVR patients (*n* = 14,933) in the Netherlands. The annual proportion of PCI procedures after TAVR increased from 0.05% in 2015 to 0.39% in 2021 (*p* < 0.001). Procedural adverse events were low. Patients treated with self‐expanding transcatheter heart valves (THVs) more frequently underwent PCI without stenting (17.8% vs. 10.1%, *p* = 0.049), though target vessel revascularization rates and all‐cause mortality were comparable. Matched patients with and without prior TAVR had similar clinical outcomes.

**Conclusions:**

The incidence of PCI after TAVR is low but increasing. Clinical outcomes are comparable between THV platforms, but self‐expanding THVs were associated with higher rates of PCI without stent implantation. The growing need for PCI after TAVR underscores the importance of coronary access in lifetime management strategies by multidisciplinary heart teams.

**Abbrevations**
CAD: coronary artery disease
MI: myocardial infarction
PCI: percutaneous coronary intervention
TAVR: transcatheter aortic valve replacement
THV: transcatheter heart valve

## 1 Introduction

Transcatheter aortic valve replacement (TAVR) is an established treatment modality for patients with severe aortic stenosis [1, 2]. Patients who are referred for TAVR frequently present with some extent of concomitant coronary artery disease (CAD), but the need for revascularization in these patients is uncertain [1, 3]. Recently, the NOTION‐3 showed that percutaneous coronary intervention (PCI) before TAVR was associated with a lower risk of a composite of death from any cause, myocardial infarction (MI), and urgent revascularization, primarily due to a reduction in MI and urgent revascularization [4]. On the contrary, other studies have failed to show beneficial effects of performing PCI before TAVR [5, 6]. Indeed, PCI before TAVR increases the risk of bleeding since it requires the use of dual antiplatelet therapy [7, 8]. As a result, several heart centers have adopted a more conservative approach, performing PCI after TAVR only when clinically indicated.

PCI after TAVR has also gained more interest since indications for TAVR continue to expand to younger patients with longer life expectancies who may more frequently require PCI after TAVR due to the natural progression of CAD. However, coronary access post‐TAVR can be technically challenging, especially in patients treated with self‐expanding transcatheter heart valves (THV) [9].

Studies on the incidence and clinical outcomes of PCI after TAVR are scarce, and primarily limited to small, single‐center registries [10, 11, 12]. More contemporary data on PCI after TAVR from larger and nationwide registries are warranted to optimize treatment and timing of concomitant CAD in patients referred for TAVR, as well as to guide lifetime management.

Therefore, the primary aim of this study was to evaluate the incidence of PCI in patients with prior TAVR, including temporal trends, and clinical outcomes in a large, nationwide cohort.

## 2 Methods

### 2.1 Patient Population and Design

Contemporary data from the Netherlands Heart Registration (NHR) were used for the current study. Detailed information on the NHR and data acquisition of the NHR has been published previously [13, 14]. In short, the NHR is a nationwide, prospective, physician‐driven, and patient‐focused quality registry aimed at maintaining and improving the quality and transparency of care for cardiac patients in the Netherlands. The registry contains demographics, baseline characteristics, procedural and clinical outcomes of all cardiac interventional procedures from Dutch heart centers, including TAVR and PCI procedures. Data are collected by participating centers (TAVR centers, *n* = 15; PCI centers, *n* = 29) and submitted to the NHR. For this retrospective multicenter study, the NHR provided an extensive database with patients who underwent PCI in the Netherlands between January 2015 and September 2021, and had a minimum follow‐up of 1 year after PCI. Additionally, the NHR provided a separate database containing procedural characteristics of TAVR procedures in patients who underwent PCI after TAVR. Patients who underwent TAVR before 2015 or those who underwent PCI concomitantly with TAVR were excluded. The study was approved by the institutional review board MEC‐U (W19.270) and conducted in agreement with the principles of the Declaration of Helsinki. A waiver for informed consent for analysis of the data of the NHR data registry was obtained.

In 2021, the registries of TAVR and PCI procedures were expanded with additional variables for patients undergoing PCI after TAVR, and were collected by most participating centers (TAVR centers, 14/15; PCI centers, 24/29). Additional variables included information on the type and size of the implanted THV, as procedural aspects of PCI procedures. Moreover, data from preprocedural computed tomography angiography (CTA) were collected, including the distances from the aortic annulus to the ostia of the left and right coronary arteries, which were used to identify patients with low coronary ostia (≤ 10 mm). Moreover, CTA‐derived aortic annulus area and perimeter were compared with the nominal area of the implanted THV to assess oversizing.

### 2.2 Study Outcomes and Definitions

The primary outcome was the incidence of PCI in patients with prior TAVR. Secondary outcomes included temporal trends, indications, and procedural and clinical outcomes of PCI in patients with prior TAVR. Lastly, clinical outcomes of patients with prior TAVR were compared to patients without prior TAVR undergoing PCI to explore the effect of THV on procedural and clinical outcomes of PCI as an exploratory analysis.

Procedural and clinical outcomes were assessed to elaborate on technical challenges in coronary cannulation in patients with prior TAVR. Procedural characteristics and outcomes were used to elaborate on technical challenges in coronary access after TAVR, including contrast fluid, fluoroscopy time, and stent implantation. PCI without stent implantation included procedures involving balloon angioplasty (plain or drug‐eluting), calcium modification without stenting, or procedures that were stopped before stent implantation. Clinical outcomes included MI, target vessel revascularization (TVR), and all‐cause mortality. MI was assessed at 30‐day follow‐up, and was defined in accordance with the definitions stated by the Task Force for the Universal Definition of MI [15, 16]. TVR was assessed at 1‐year follow‐up and defined as a recurrent revascularization of the same vessel as treated during the index PCI. Information on TVR was available for patients treated after 2017. Data on target lesion revascularization, which is defined as a recurrent revascularization of the same lesion as treated during the index PCI, became available only in 2019. Mortality status was obtained by checking the regional municipal administration registration at 1‐year follow‐up.

### 2.3 Statistical Analysis

Continuous variables were summarized as means and standard deviations or medians with interquartile ranges (IQR) and were compared with the Student *t*‐test and or with the Mann−Whitney *U* test, as appropriate. Categorical were presented as numbers and percentages, and tested with the chi‐square test or Fisher's exact test, as appropriate. Incidence of PCI in patients with prior TAVR was assessed, and trends in annual incidence of PCI in patients with prior TAVR were explored using the chi‐square test for trend. Indications for PCI were explored and categorized into elective and urgent indications, where urgent indications included ST‐elevated MI (STEMI) and non‐STEMI (NSTEMI). Indications were also assessed for three different time windows between TAVR and PCI, as defined in accordance with definitions used by the Valve Academic Research Consortium (VARC)‐3: 0–30 days, 31–365 days, and more than 365 days, respectively [17]. Procedural and clinical outcomes were compared for patients treated with balloon‐expandable and self‐expanding THV. Moreover, univariate logistic regression was used to identify predictors for unfavorable procedural and clinical outcomes of PCI in patients with prior TAVR, considering preprocedural CTA measurements and procedural characteristics of the TAVR procedure. Predictors with *p* < 0.10 were simultaneously entered in a multivariable logistic regression model.

For the comparison of patients with and without prior TAVR, we used propensity score matching, adjusting for age, sex, previous MI, previous cardiac interventions, diabetes mellitus, chronic kidney disease, indication, and access for PCI, number of treated vessels, and presence of chronic total occlusion, without imputation for missing values. For each patient who underwent PCI after TAVR, a corresponding patient without prior TAVR was selected on the basis of the nearest propensity score using the one‐to‐one nearest neighbor method (using a caliper of 0.2 standard deviations of the propensity score on the logit scale) without replacement. Time‐to‐event curves were established for TVR and mortality using the Kaplan–Meier method. Hazard ratios (HR) with 95% confidence intervals (CI) were calculated for TVR using Fine‐Gray models after adjustment for the competing risk of mortality. Patients without prior TAVR were used as the reference group. No formal statistical testing was performed for all‐cause mortality within the matched cohort due to the risk of unmeasured confounding for mortality within the used variables.

All statistical tests were two‐tailed, and *p* < 0.05 was considered statistically significant. Statistical analyses were performed using SPSS (version 28.0 for Windows, IBM Corp) and R for propensity score matching (version 4.3.2).

## 3 Results

### 3.1 Patient Population

**Figure 1:**
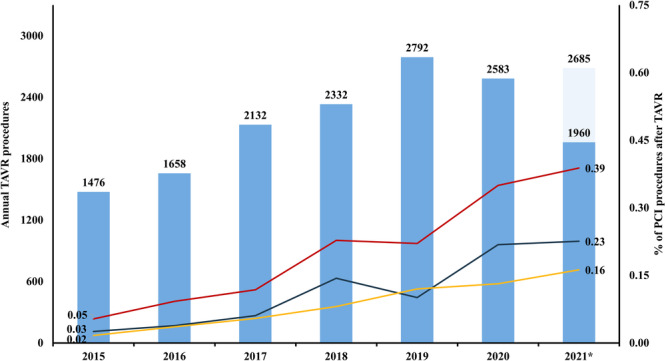
Annual number of patients undergoing TAVR, and proportion of PCI procedures performed after TAVR. PCI = percutaneous coronary intervention, TAVR = transcatheter aortic valve replacement. *2021: only Q1–Q3 included in current study (*n* = 1960, total *n* in 2021 = 2685). [Color figure can be viewed at wileyonlinelibrary.com]

Between January 2015 and September 2021, a total of 216,813 patients underwent PCI in the Netherlands. Among them, 419 (0.19%) patients had previously undergone TAVR, representing 2.81% of all patients who underwent TAVR within the same period. Mean age of patients with prior TAVR was 79.0 ± 7.1 years, the majority (60.6%) were male, and the median European System for Cardiac Operative Risk Evaluation (EuroSCORE) II at time of TAVR was 3.32% (IQR: 1.97%–6.03%). The majority of patients underwent TAVR with a balloon‐expandable THV (57.1%). Patients without prior TAVR were younger, more frequently female, and had fewer comorbidities. Table 1 presents baseline characteristics of the included cohort. The annual proportion of PCI procedures performed in patients with prior TAVR increased over time: from 0.05% in 2015 to 0.39% in 2021 (*p* for trend < 0.001, Figure 1).

**Table 1:** Baseline characteristics of patients with and without prior TAVR who underwent PCI.

	With prior TAVR (*n* = 419)	Without prior TAVR (*n* = 216,394)	*p* value
*Demographics*			
Age (years)	79.0 ± 7.1	66.4 ± 11.6	< 0.001
Women	165/419 (39.4%)	61,473/216,392 (28.4%)	< 0.001
*Medical history*			
Prior MI	137/407 (33.7%)	34,950/194,541 (18.0%)	< 0.001
Prior PCI	227/382 (59.4%)	27,297/152,639 (17.9%)	< 0.001
Prior CABG	103/407 (25.3%)	16,166/198,337 (8.2%)	< 0.001
Diabetes mellitus	137/404 (33.9%)	40,670/194,507 (20.9%)	< 0.001
Chronic kidney disease (eGFR < 60)	206/396 (52.0%)	42,373/184,722 (22.9%)	< 0.001
Chronic dialysis	9/396 (2.3%)	708/184,722 (0.4%)	< 0.001
LVEF ≥ 50%	129/243 (53.1%)	52,741/81,580 (64.6%)	< 0.001
*Procedural characteristics PCI*			
Reason for PCI (%)			< 0.001
Elective	174/407 (42.8%)	68,019/196,904 (34.5%)	
NSTEMI	177/407 (43.5%)	66,492/196,904 (33.8%)	
STEMI	56/407 (13.8%)	62,393/196,904 (31.7%)	
Out‐of‐hospital cardiac arrest (%)	14/408 (3.4%)	7,291/197,558 (3.7%)	0.781
Cardiogenic shock (%)	12/405 (3.0%)	5587/197,529 (2.8%)	0.870
*Treated vessel (%)*			
Left main coronary artery	46/364 (12.6%)	6,273/145,919 (4.3%)	< 0.001
Left anterior descending artery	135/364 (37.1%)	72,341/145,919 (49.6%)	< 0.001
Left circumflex artery	106/364 (29.1%)	36,834/145,919 (25.2%)	0.089
Anterolateral coronary artery	7/364 (1.9%)	3,268/145,919 (2.2%)	0.684
Right coronary artery	108/364 (29.7%)	51,942/145,919 (35.6%)	0.018
Arterial bypass graft	3/364 (0.8%)	252/145,919 (0.2%)	0.003
Venous bypass graft	30/364 (8.2%)	2,408/145,919 (1.7%)	< 0.001
Multivessel procedure (%)	80/364 (22.0%)	26,016/145,919 (17.8%)	0.039
Chronic total occlusion (%)	14/407 (3.4%)	9,866/197,292 (5.0%)	0.149
Radial access (%)	257/366 (70.2%)	118,070/137,524 (85.9%)	< 0.001
Procedural characteristics TAVR*			
EuroSCORE II	3.32 (1.97–6.03)	—	
Prior SAVR	22/402 (5.5%)	—	
Femoral access	290/395 (73.4%)	—	
Balloon‐expandable THV	213/373 (57.1%)	—	
Type of THV			
Sapien/Sapien XT/Sapien 3 (Ultra)	210/373 (56.3%)	—	
CoreValve/Evolut R/Evolut PRO	118/373 (31.6%)	—	
Acurate/Acurate Neo	16/373 (4.3%)	—	
Portico	2/373 (0.5%)	—	
Other	27/373 (7.2%)	—	
Valve size(mm)	26 (26–29)	—	

Abbreviations: CABG = coronary artery bypass graft, LVEF = left ventricular ejection fraction, MI = myocardial infarction, NSTEMI = non‐ST‐elevated myocardial infarction, PCI = percutaneous coronary intervention, STEMI = ST‐elevated myocardial infarction, TAVR = transcatheter aortic valve replacement, THV = transcatheter heart valve.

### 3.2 Timing and Indications of PCI in Patients With Prior TAVR

**Figure 2:**
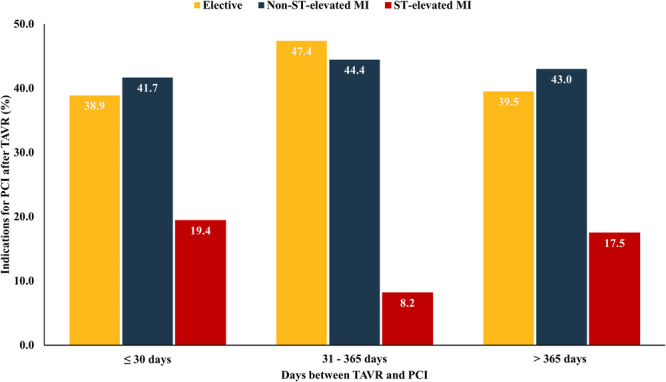
Indications for PCI after TAVR in different time periods after TAVR. PCI = percutaneous coronary intervention, TAVR = transcatheter aortic valve replacement. [Color figure can be viewed at wileyonlinelibrary.com]

Median time between TAVR and PCI was 347 (IQR: 119–817) days. A total of 217 (51.2%) patients underwent PCI within the first year after TAVR, including 39 (9.3%) patients who underwent PCI within the first month after TAVR. NSTEMI was the predominant reason for PCI after TAVR (Table 1). Elective PCI after TAVR was performed in 174 (42.8%) patients, with 54.6% of these elective procedures performed within the first year after TAVR. The distribution of indications for PCI within different time periods after TAVR did not change (*p* = 0.082, Figure 2). In patients without prior TAVR, elective indications (34.5%) were the predominant reason for PCI. Also, STEMI was more frequently reported as an indication for PCI compared to patients with prior TAVR (13.8% vs. 31.7%, *p* < 0.001).

### 3.3 Characteristics of PCI After TAVR

Patients who underwent PCI after TAVR more frequently presented with CAD involving either multivessel or left main CAD compared with patients without prior TAVR (22.0% vs. 17.8%, *p* = 0.039). Also, the femoral artery was more commonly used as primary access for PCI in patients with prior TAVR compared to patients without prior TAVR (29.2% vs. 14.1%, *p* < 0.001). In patients with prior TAVR, the amount of contrast fluid used during PCI was 150 (IQR: 100–200) cc and fluoroscopy time was 16.4 (IQR: 10.0–22.9) minutes. The amount of contrast fluid and fluoroscopy time did not change over time (*p* = 0.600 and *p* = 0.750, respectively, Supporting Information S1: Figure 1). There were no differences between patients who underwent TAVR with different THV platforms. Elective PCI procedures were associated with more contrast volume compared to urgent PCI procedures (150 [IQR 100–220] vs. 139 [IQR 100–180], *p* = 0.043), but fluoroscopy time was similar (18 [IQR 11.5–24.7] vs. 14.8 [IQR 9.6–22.4], *p* = 0.075).

### 3.4 Procedural and Clinical Outcomes of PCI After TAVR

**Figure 3:**
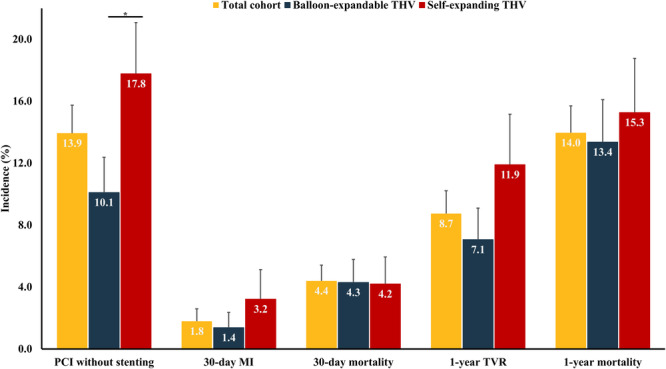
Procedural outcomes of PCI after TAVR in the total cohort, and in patients treated with balloon‐expandable and self‐expanding THVs. MI = myocardial infarction, PCI = percutaneous coronary intervention, TAVR = transcatheter aortic valve replacement, THV = transcatheter heart valve, TVR = target vessel revascularization. *p < 0.05. [Color figure can be viewed at wileyonlinelibrary.com]

Figure 3 displays procedural and clinical outcomes of patients who underwent PCI after TAVR. PCI was prematurely ended in 4 (1.1%) patients. PCI without stenting was performed in 51 (13.9%) patients with prior TAVR. MI within 30 days was reported in 5 (1.8%) patients. TVR was performed in 32 (8.7%) patients, involving target lesion revascularization in at least 50% of patients. All‐cause mortality within 30 days was reported in 18 (4.4%) patients, including 5 patients who died on the day of PCI. Additionally, a higher incidence of PCI without stenting was observed among patients who underwent TAVR with self‐expanding THVs compared to patients treated with balloon‐expandable THVs (17.8% vs. 10.1%, *p* = 0.049). Thirty‐day MI, 30‐day and 1‐year mortality, and 1‐year TVR were comparable between patients who underwent TAVR with balloon‐expandable and self‐expanding THV (Figure 3).

### 3.5 Predictors of Difficulties and Unfavorable PCI Outcomes After TAVR

Additional data on pre‐TAVR CTA measurements were available in 222 (53%) patients. Mean annular area was 480 ± 96 mm^2^. Mean height between the aortic annulus and the ostium of the target vessel was 16.1 ± 3.8 mm in patients with prior TAVR undergoing PCI of the right coronary artery, whereas mean height was 14.6 ± 4.2 mm in patients undergoing PCI in the left coronary artery. Based on the distance between the aortic annulus and ostium of the target vessel, 11 (4.8%) patients were found to have an increased risk of interference of THV with coronary ostia within 10 millimeters of the annular plane. As displayed in Supporting Information S1: Table 1, no predictors of increased risk of unfavorable clinical outcomes were identified among preprocedural CTA measurements and TAVR characteristics. Additionally, there was no correlation between coronary height and the amount of contrast fluid, nor between coronary height and fluoroscopy time.

### 3.6 Clinical Outcomes in Matched Patients With and Without Prior TAVR

**Figure 4:**
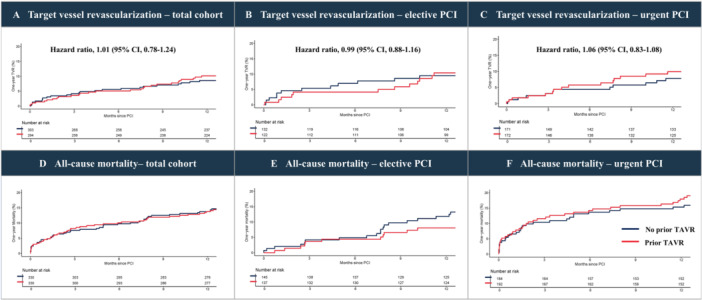
Time‐to‐event curves for TVR and all‐cause mortality in matched cohort. Shown are Kaplan‐Meier estimates of the rate of TVR in the total cohort (A), for patients undergoing elective PCI (B), and for patients undergoing urgent PCI (C), as well as for all‐cause mortality in the total cohort (D), for patients undergoing elective PCI (E), and for patients undergoing urgent PCI (F). Hazard ratios and 95% confidence intervals are displayed for TVR. PCI = percutaneous coronary intervention, TAVR = transcatheter aortic valve replacement, TVR = target vessel revascularization. [Color figure can be viewed at wileyonlinelibrary.com]

The propensity‐matched cohort consisted of 330 pairs of patients undergoing PCI, with and without prior TAVR. Balance of various patient‐ and PCI characteristics before and after adjustment is displayed in Supporting Information S1: Table 2 and Supporting Information S1: Figure 2. The incidence of PCI without stenting was similar between matched patients with and without prior TAVR (11.5% vs. 9.7%, *p* = 0.448). Patients with prior TAVR reported similar rates of TVR during 1‐year follow‐up compared to matched patients without prior TAVR (9.2% vs. 7.9%; HR: 1.16; 95% CI, 0.67–2.01, Figure 4A). Additionally, no differences were found in the rate of TVR between matched patients with and without prior TAVR for elective and urgent PCI procedures (Figure 4B,C). All‐cause mortality after PCI in matched patients with and without TAVR is displayed in Figure 4D, along with all‐cause mortality after elective and urgent PCI procedures in Figure 4E,F.

## 4 Discussion

The current study on PCI in patients with prior TAVR has revealed several key findings. First, the incidence of patients undergoing PCI after TAVR is low, but the number of patients requiring PCI is increasing. Second, the majority of patients underwent PCI after TAVR for urgent indications, primarily NSTEMI. Third, procedural and clinical outcomes are comparable for various THV platforms, but PCI procedures without stent implantation were more frequent among patients who were treated with self‐expanding THVs. Fourth, patients who underwent PCI after TAVR demonstrated similar procedural outcomes as matched patients without prior TAVR (Central Illustration 1).

**Central Illustration 1:**
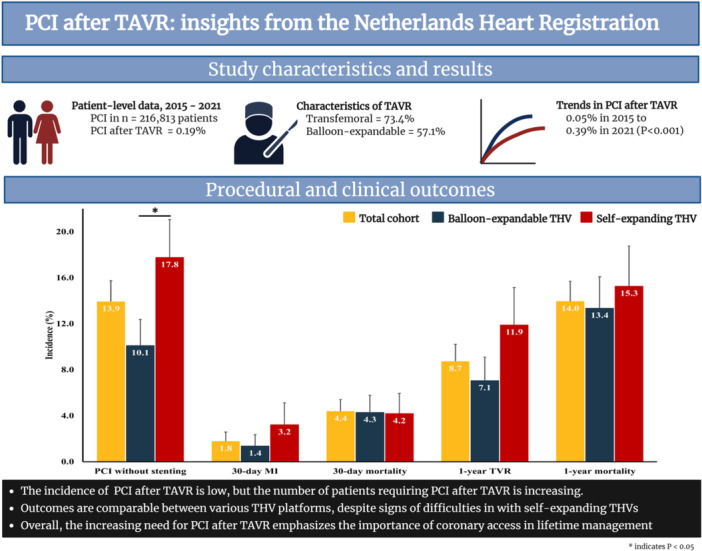
PCI after TAV: insights from the nationwide Netherlands Heart Registration. [Color figure can be viewed at wileyonlinelibrary.com]

### 4.1 Incidence and Indications of PCI After TAVR

CAD is a frequent bystander in patients with severe aortic valve stenosis, with its prevalence reported in up to half of those referred for TAVR [3]. The treatment of concomitant CAD in patients undergoing TAVR has evolved over the last decade. The first pivotal trials excluded patients with untreated concomitant CAD, highlighting the importance of preprocedural revascularization before TAVR [18, 19]. This strategy was recently confirmed by the NOTION‐3 trial as PCI in patients with important concomitant CAD undergoing TAVR was associated with a lower risk of a composite of death from any cause, MI, or urgent revascularization compared with conservative treatment [4]. However, this benefit was primarily driven by reductions in MI and urgent revascularization, with no difference in mortality observed between patients undergoing TAVR with and without prior PCI. Interestingly, in recent years, a TAVR‐only strategy has been increasingly adopted in patients with concomitant CAD, considering the risks associated with PCI and the increased bleeding risk from dual antiplatelet therapy during TAVR. Our nationwide study is the first to investigate recent trends in the incidence of PCI after TAVR, using information from more than 200,000 contemporary patients undergoing PCI in the Netherlands between 2015 and 2021. Our results indicate that only 0.19% of these patients had previously undergone TAVR, corresponding to 2.81% of all patients who underwent TAVR in Dutch heart centers within the same period. This finding is in line with results from a Swedish registry, whereas results from a French nationwide registry showed that the incidence of PCI after TAVR varied between 5.4% for patients treated with balloon‐expandable THVs and 3.6% for those treated with self‐expanding THVs [10, 20]. Results from older, and primarily single‐center studies indicated varying incidences between 0.9% and 5.84% [11, 12, 21]. More importantly, we are the first to show that the incidence of PCI in patients with prior TAVR increases over time: from 0.05% in 2015 to 0.39% in 2021. Although this finding may be at least partially attributable to the increasing number of patients undergoing TAVR, it nonetheless underscores the growing importance of incorporating post‐TAVR coronary access in preprocedural planning by multidisciplinary heart teams.

Most of the patients in our nationwide registry required PCI after TAVR for urgent reasons, primarily NSTEMI. Interestingly, our study found that the incidence of PCI for STEMI was lower among patients with prior TAVR compared to those without, which may reflect a greater reluctance to perform interventions in these circumstances. Nonetheless, more than 40% of patients underwent PCI in an elective setting, with the vast majority performed within the first year after TAVR. Although our observations are consistent with the findings of the REVIVAL registry, comprising 133 patients who underwent PCI after TAVR at major heart centers across Europe, they are in contrast to those of the France‐TAVI registry, which reported that coronary events post‐TAVR are predominantly attributable to chronic coronary syndromes [20, 21]. These variances may be attributed to differences in important patient characteristics, such as the extent of pre‐TAVR CAD and varying revascularization strategies in patients referred for TAVR.

### 4.2 Clinical Outcomes and Predictors for Difficulties in PCI After TAVR

With a growing number of patients requiring PCI after TAVR, multidisciplinary heart teams and interventionalists should be informed about procedural difficulties and clinical outcomes. Whereas numerous studies have shown important predictors of impaired coronary access after TAVR, data on clinical outcomes are scarce [9, 22, 23]. Our study found that the incidence of adverse events at short‐term follow‐up is low, suggesting that PCI after TAVR is safe. The latter is confirmed by the results of our analysis in matched patients with and without prior TAVR, revealing similar results. However, we observed differences between patients treated with balloon‐expandable and self‐expanding THVs. First, PCI without stent implantation was more frequently observed in patients with self‐expanding THVs. Moreover, the incidence of recurrent PCI of index lesions was numerically higher among patients with self‐expanding THVs. On the contrary, fluoroscopy time and the amount of contrast fluid were similar among the two platforms. Nonetheless, our findings suggest that PCI after TAVR may be more difficult to perform in patients who were treated with self‐expanding THVs compared to patients who underwent TAVR with balloon‐expandable THV. This observation is in accordance with studies that focused on the feasibility of coronary access after TAVR, including procedural and technical. The ALIGN‐ACCESS study revealed that THVs with a self‐expanding design are associated with higher rates of impaired coronary access [22], whereas results from the REACCESS studies emphasize that this association is predominantly found in patients treated with Evolut THVs [9, 23]. The latter are primarily characterized by a smaller cell design, making coronary cannulation more challenging than with other self‐expanding THV platforms that feature larger cell designs. To overcome these limitations, the adoption of commissural alignment, especially in THVs with a supra‐annular design, increases the rates of successful coronary access after TAVR [22]. Additionally, these technical studies identified several predictors of impaired coronary access based on preprocedural CTA measurements, including the oversizing of the THV relative to the sinus of Valsalva [9]. In our nationwide study, we did not observe any association between collected preprocedural CTA measurements and unfavorable clinical outcomes. Overall, these findings suggest that the presence of a self‐expanding THV may be the most important concern for difficulties during PCI after TAVR.

### 4.3 Future Implications and Perspectives

Our study demonstrates that PCI after TAVR is safe but stresses the need for comprehensive lifetime management as the incidence of PCI after TAVR is increasing. This observed trend in the need for post‐TAVR PCI will be augmented by expanding indications toward younger patients with longer life expectancy [24]. Additionally, multiple ongoing randomized trials are investigating both the need for PCI in patients undergoing TAVR as well as the optimal timing. The Dutch PRO‐TAVI trial (PCI Before Transcatheter Aortic Valve Implantation; NCT05078619) is investigating the non‐inferiority of deferral of routine PCI in patients undergoing TAVR [25]. The Italian FAITAVI trial (Functional Assessment in Transcatheter Aortic Valve Implantation; NCT03360591) aims to investigate the optimal PCI strategy in patients with concomitant CAD undergoing TAVR, evaluating both timing as well as the use of physiology [26]. Additionally, the COMPLETE TAVR trial (Staged Complete Revascularization for CAD vs. Medical Management Alone in Patients With AS Undergoing TAVR; NCT04634240), including 4000 US and Canadian patients, will investigate the importance of complete revascularization in patients who successfully underwent TAVR with a balloon‐expandable THV. Alongside the various trials on revascularization strategies in patients undergoing TAVR, antithrombotic treatment strategies have recently gained attention (Personalized, CT‐guided Antithrombotic Therapy vs. Lifelong Single Antiplatelet Therapy to Reduce Thromboembolic and Bleeding Events in Non‐atrial Fibrillation Patients After Transcatheter Aortic Valve Implantation (POP ATLANTIS); NCT0616870). These strategies may play a key role in valve durability, and with the introduction of the neo‐sinus phenomenon, they could also influence coronary access after TAVR [27].

Taken together, the need for coronary access after TAVR is becoming a key issue for multidisciplinary heart teams, and our findings as well as those from recent technical studies should be incorporated into lifetime management strategies for future TAVR patients.

### 4.4 Limitations

First, the retrospective design of our registry‐based study has its well‐known inherent limitations. Second, the number of patients who underwent unsuccessful coronary angiography without subsequent PCI was not documented, and the registry did not contain information on the use of intracoronary imaging or physiological assessment, nor information on antithrombotic therapy. Third, MI was assessed only at 30‐day follow‐up; data on TVR were only available since 2019, without additional information on in‐stent restenosis without repeat revascularization. Importantly, clinical outcomes were site‐reported without central adjudication. Fourth, only a few patients in our study had previously undergone valve‐in‐valve procedures, so our findings should be extrapolated with caution to this group, who are known to be at higher risk for impaired coronary access. Lastly, the results of our hypothesis‐generating comparison between patients with and without prior TAVR should be interpreted with caution due to the inherent limitations of comparisons using propensity score matching.

## 5 Conclusion

The incidence of patients undergoing PCI after TAVR is low, but the number of patients requiring PCI after TAVR is increasing. Procedural and clinical outcomes of patients undergoing PCI after TAVR are comparable between various THV platforms, despite signs of difficulties in patients treated with self‐expanding THVs. Overall, the increasing need for PCI after TAVR emphasizes that coronary access should play a key role in lifetime management strategies for future TAVR patients.

## Conflicts of Interest

Dr. Delewi received educational grants from Abiomed, Boston Scientific, Meril Life Science, Sanofi, Novartis, Amgen, and Edwards Lifesciences. The other authors declare no conflicts of interest.

## Supporting information


**Supplementary Figure S1:** Contrast fluid and fluoroscopy time during PCI after TAVR. **Supplementary Figure S2:** Love plot displaying covariate balance before and after adjustment. **Supplementary Table S1:** Overview of characteristics for adjusted comparison of patients with and without prior TAVR who underwent PCI. **Supplementary Table S2:** Results of logistic regression analysis to explore potential predictors for suboptimal outcomes of PCI in patients with prior TAVR.
