# Supplementary material for: Nationwide Analysis of PCI After TAVR From the Netherlands Heart Registration
Source: Catheter Cardiovasc Interv. 2025 Dec 10;107(3):824–32. doi: 10.1002/ccd.70428 (PMC12902725; doi:10.1002/ccd.70428)
Supplement: Supplementary file 1 — Supplementary Figure S1: Contrast fluid and fluoroscopy time during PCI after TAVR. Supplementary Figure S2: Love plot displaying covariate balance before and after adjustment. Supplementary Table S1: Overview of characteristics for adjusted comparison of patients with and without prior TAVR who underwent PCI. Supplementary Table S2: Results of logistic regression analysis to explore potential predictors for suboptimal outcomes of PCI in patients with prior TAVR. [file CCD-107-824-s001.docx]

**PCI after TAVR – Supplemental Appendix**

1. Supplementary Table S1. Overview of characteristics for adjusted comparison of patients with and without prior TAVR who underwent PCI.
2. Supplementary Table S2. Results of logistic regression analysis to explore potential predictors for suboptimal outcomes of PCI in patients with prior TAVR.
3. Supplementary Figure S1. Contrast fluid and fluoroscopy time during PCI after TAVR.
4. Supplementary Figure S2. Love plot displaying covariate balance before and after adjustment.

**Supplementary Table S1. Results of logistic regression analysis to explore potential predictors for suboptimal outcomes of PCI in patients with prior TAVR.**

| **Variable** | **OR (95% CI)**  **PCI without stenting** | **OR (95% CI)**  **30-day MI** | **OR (95% CI)**  **1-year TVR** |
| --- | --- | --- | --- |
| Prior SAVR | 1.48 (0.33-6.58) P=0.607 | P=1.000 | 0.55 (0.72-4.29) P=0.571 |
| Distance to RCA (mm) | 0.93 (0.85-1.02) P=0.116 | 1.15 (0.95-1.38) P=0.145 | 1.12 (1.01-1.24) P=0.030 |
| Distance to LCA (mm) | 0.95 (0.87-1.04) P=0.288 | 1.07 (0.88-1.30) P=0.523 | 0.95 (0.83-1.07) P=0.386 |
| Aortic annulus (mm) | 0.99 (0.94-1.04) P=0.611 | 1.03 (0.89-1.19) P=0.733 | 1.01 (0.95-1.08) P=0.780 |
| THV, under- or oversizing | 1.01 (0.98-1.03) P=0.570 | 1.01 (0.92-1.10) P=0.898 | 1.02 (0.99-1.06) P=0.209 |
| THV, self-expanding design | 0.68 (0.08-5.93) P=0.723 | 2.38 (0.39-14.54) P=0.347 | 1.78 (0.82-3.88) P=0.148 |
| THV, Evolut | 0.60 (0.32-1.15) P=0.127 | 1.62 (0.27-9.93) P=0.599 | 1.38 (0.62-3.06) P=0.432 |
| TAVR procedure in 2021 | 0.61 (0.14-2.60) P=0.505 | P=1.000 | 1.00 (0.10-10.5) P=1.000 |

1. *LCA= left coronary artery; MI= myocardial infarction; PCI= percutaneous coronary intervention; RCA= right coronary artery; SAVR= surgical aortic valve replacement; TAVR= transcatheter aortic valve replacement; THV=transcatheter heart valve; TVR=target vessel revascularization.*

**Supplementary Table S2. Overview of characteristics for adjusted comparison of patients with and without prior TAVR who underwent PCI.**

|  | **With prior TAVR**  **(n= 330)** | **Without prior TAVR**  **(n= 330)** | **SMD**  **before matching** | | **SMD**  **after matching** | **P value** |
| --- | --- | --- | --- | --- | --- | --- |
| **Demographics** |  |  | |  |  |  |
| Age (years) | 78.8 ± 6.7 | 79.1 ± 6.6 | | 1.09 | -0.05 | 0.539 |
| Women | 119 (36.1%) | 122 (37.0%) | | 0.24 | -0.02 | 0.808 |
| **Medical history** |  |  | |  |  |  |
| Prior MI | 110 (33.3%) | 107 (32.4%) | | 0.41 | 0.02 | 0.804 |
| Prior PCI | 195 (59.1%) | 187 (56.7%) | | 1.08 | 0.05 | 0.528 |
| Prior CABG | 82 (24.8%) | 92 (27.9%) | | 0.63 | -0.07 | 0.377 |
| Diabetes mellitus | 113 (34.2%) | 112 (33.9%) | | 0.32 | 0.01 | 0.935 |
| Chronic kidney disease (eGFR <60) | 168 (50.9%) | 164 (49.7%) | | 0.69 | 0.02 | 0.919 |
| Chronic dialysis | 9 (2.7%) | 6 (1.8%) | | 0.27 | 0.06 | 0.451 |
| LVEF ≥ 50% | 106/198 (53.5%) | 101/193 (52.3%) | | 0.27 | -0.05 | 0.910 |
| **Procedural characteristics PCI** |  |  | |  |  |  |
| Reason for PCI (%) |  |  | | -0.27 | -0.003 | 0.796 |
| Elective | 137/329 (41.6%) | 137/326 (42.0%) | |  |  |  |
| NSTEMI | 142/329 (43.2%) | 134/326 (41.1%) | |  |  |  |
| STEMI | 50/329 (15.2%) | 55/326 (16.9%) | |  |  |  |
| Out-of-hospital cardiac arrest (%) | 10 (3.0%) | 8 (2.4%) | | -0.01 | 0.04 | 0.633 |
| Cardiogenic shock (%) | 11 (3.3%) | 11 (3.3%) | | 0.01 | 0.00 | 1.000 |
| Treated vessel (%) |  |  | |  |  |  |
| Left main coronary artery | 43 (13.0%) | 34 (10.3%) | | 0.41 | 0.08 | 0.275 |
| Left anterior descending artery | 120 (36.4%) | 148 (44.8%) | | -0.25 | -0.17 | 0.026 |
| Left circumflex artery | 96 (29.1%) | 67 (20.3%) | | 0.09 | 0.20 | 0.009 |
| Anterolateral coronary artery | 5 (1.5%) | 3 (0.9%) | | -0.02 | 0.06 | 0.477 |
| Right coronary artery | 103 (31.2%) | 107 (32.4%) | | -0.12 | -0.02 | 0.738 |
| Arterial bypass graft | 3 (0.9%) | 2 (0.6%) | | 0.16 | -0.03 | 0.653 |
| Venous bypass graft | 26 (7.9%) | 29 (8.8%) | | 0.52 | -0.03 | 0.673 |
| Multivessel procedure (%) | 75 (22.7%) | 69 (20.9%) | | 0.11 | 0.04 | 0.572 |
| Chronic total occlusion (%) | 13 (3.9%) | 13 (3.9%) | | -0.07 | 0.00 | 1.000 |
| Radial access (%) | 231 (70.0%) | 232 (70.3%) | | 0.44 | -0.01 | 0.796 |

*CABG= coronary artery bypass graft; LVEF= left ventricular ejection fraction; NSTEMI= non-ST-elevated myocardial infarction; PCI= percutaneous coronary intervention; SAVR= surgical aortic valve replacement; SMD = standardized mean difference; STEMI= ST-elevated myocardial infarction; TAVR= transcatheter aortic valve replacement.*

**Supplementary Figure S1. Contrast fluid and fluoroscopy time during PCI after TAVR.**

*
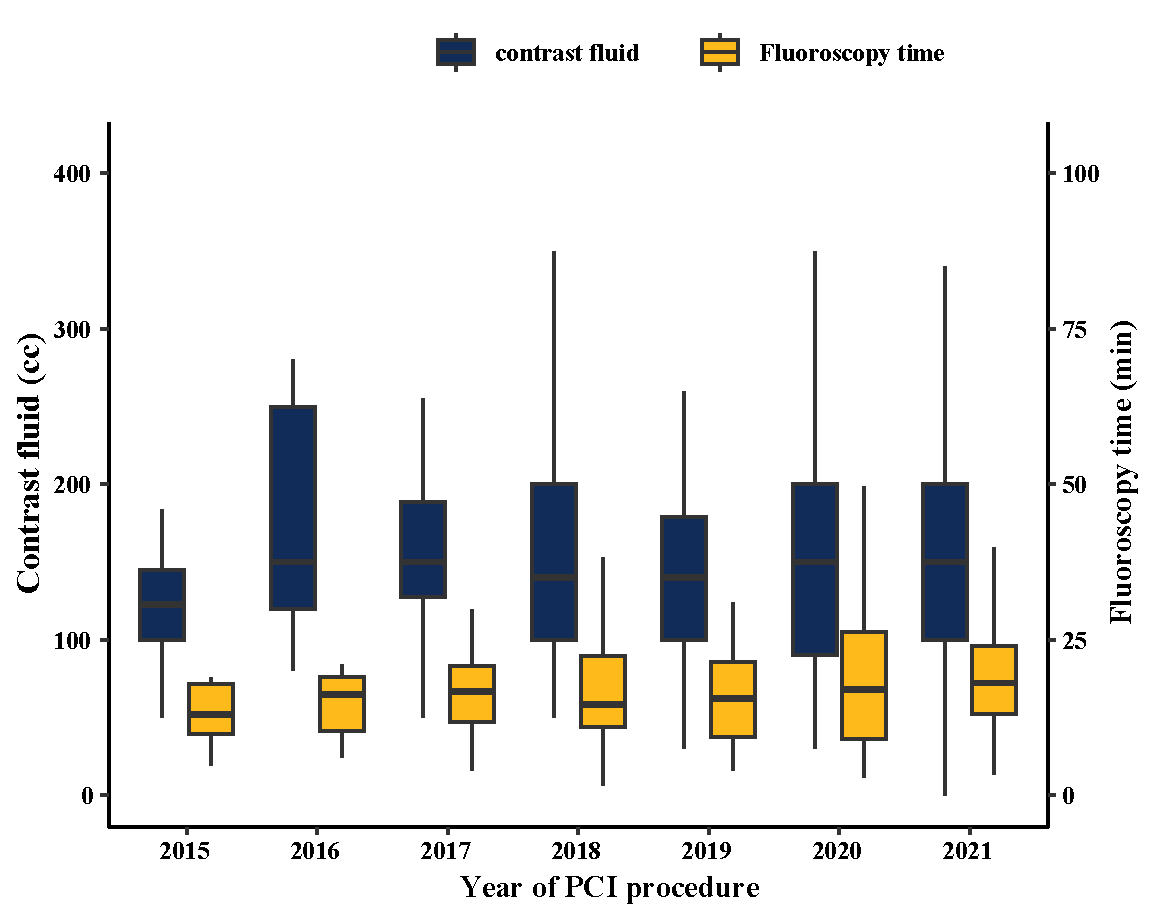
*

*PCI= percutaneous coronary intervention; TAVR= transcatheter aortic valve replacement.*

**Supplementary Figure S2. Love plot displaying covariate balance before and after adjustment.**

**
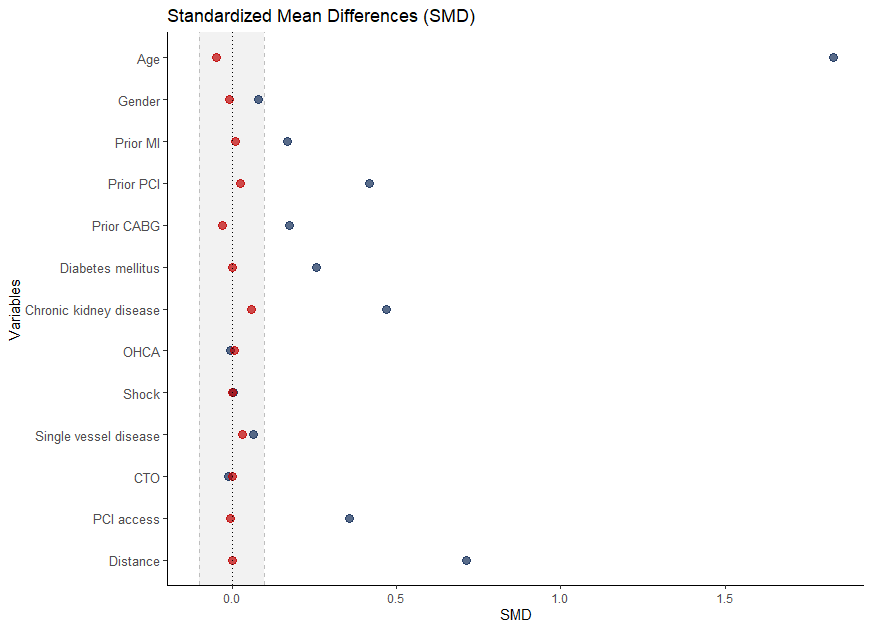
**

*CABG= coronary artery bypass graft; CTO= chronic total occlusion; MI= myocardial infarction; OHCA= out-of-hospital cardiac arrest; PCI= percutaneous coronary intervention*
